# OP17 Methods To Measure The Environmental Impacts Of Health Technologies And Include Them In Economic Evaluations: A Scoping Review

**DOI:** 10.1017/S0266462324000801

**Published:** 2025-01-07

**Authors:** Vittoria Ardito, Helen Banks, Niccolò Cusumano, Rosanna Tarricone

## Abstract

**Introduction:**

The environmental footprint of the healthcare sector is widely acknowledged and increasingly impacting decision-making in health policy. This study aims to explore what methods and metrics have been proposed to measure environmental impact of health technologies, and to investigate the feasibility and consequences of its inclusion in economic evaluations. This study is part of a larger Horizon Europe project, HI-PRIX.

**Methods:**

We conducted a PRISMA-ScR-guided scoping review of the scientific publications in PubMed, Web of Science, and Scopus between 2013 and November 2023. For the grey literature, the international HTA database and HTA agencies websites were searched to identify ongoing or published HTA dossiers. The search strategy was developed around two concepts: environment and health technology assessment. Any study or report that described a conceptual framework, methodology, or approach used or theorized to integrate environmental impacts of health technologies in an HTA was considered eligible for inclusion.

**Results:**

Starting from 12,336 records, 16 scientific publications and 6 HTA dossiers were included for analysis. One-third of the contributions were published in 2023 and were mostly opinions, editorials, reviews, or economic evaluations. Lifecycle assessment was considered the preferred approach to investigate environmental impacts, yet difficulties exist; for example, methodologies for measuring greenhouse gas emissions are established, while for other dimensions are under development. Nevertheless, methods to incorporate environmental impact into economic models appear viable. About half of the papers analyzed cost–utility analysis, multiple-criteria decision analysis, and cost–benefit analysis as potential approaches. Fewer papers addressed budget impact, cost-effectiveness analysis, or other approaches.

**Conclusions:**

Incorporating the environmental impact of health technologies in economic evaluations is under development, but consensus is still lacking on appropriate, feasible methodologies for its uptake. Before considering it in pricing or reimbursement-related decisions, the preferred method to maximize utility levels of different stakeholders (e.g., HTA agencies, industry) needs clarification.

